# Complications of Uterine Fibroids and Their Management

**DOI:** 10.1155/2012/932436

**Published:** 2012-03-19

**Authors:** Horace Fletcher, Celia Burrell, Bharat Bassaw, Earlando Thomas

**Affiliations:** ^1^Department of Obstetrics and Gynaecology, The University of the West Indies, Kingston 7 Mona, Jamaica; ^2^Department of Obstetrics and Gynecology, Barking, Havering and Redbridge University Hospital, Queens Hospital, Romford, Essex RM7 0AG, UK; ^3^The University of the West Indies, Mount Hope, St. Augustine, Trinidad and Tobago; ^4^Rochester General Hospital, 1425 Portland Avenue, Rochester, NY 14621, USA

Uterine fibroids are the most common gynaecological tumours found in women. These tumours are said to be present in 80% of Caucasian women and are even more common in women of African origin. In 50% of women with fibroids symptoms usually occur, leading to complications from the presence of these fibroids. Although they are mainly a problem in the reproductive years, there are reports of problems from fibroids in postmenopausal women.

The management of patients with fibroids is quite variable including conservative oral and parenteral treatment, conservative and radical surgery, and newer techniques such as uterine artery embolisation and radiofrequency ablation. All of these techniques can also cause complications for the patient, and as a result careful evaluation of patients must be done to decide on the correct treatment option for a particular patient.

The focus of this special issue is to highlight the complications which are caused by fibroids and also those which can arise as a result of the treatment options available. This is intended to assist clinicians and their patients in making informed decisions about the management of this common problem.

 The journal is the effort of a truly international group of clinicians giving varied experiences from across the world.

The paper entitled “*Complications of uterine fibroids and their management*” is from Jamaica and outlines the entire spectrum of complications of fibroids and their treatment. This is a good review of the modern methods available and should be helpful to clinicians to assist them in counseling their patients.

The paper entitled “*Innovative oral therapy for uterine leiomyomas*” is a collaborative effort of clinicians from Egypt and the United States of America (USA) reviewing the use of oral therapy for the treatment of fibroids. This paper gives an update on the modern use of drugs to treat fibroids to avoid surgery.

The paper entitled “*Surgical management of uterine fibroids at Aminu Kano Teaching Hospital*” is from Nigeria, and this paper discusses use of myomectomy versus abdominal hysterectomy in their setting. The authors have outlined their experience and given the reader an idea of their methodology and complication rates comparing the two procedures.

The paper entitled “*Intrauterine adhesions following conservative treatment of uterine fibroids*” is from Sweden and Spain discussing the complication of intrauterine adhesions reminding readers how to diagnose and treat this often forgotten complication of surgical treatment of fibroids.

The paper entitled “*Indications and outcomes of uterine artery embolization in patients with uterine leiomyomas*” is from Japan, and the paper entitled “*Complications associated with uterine artery embolisation for fibroids*” is from the United Kingdom. Both papers discuss their experience with uterine artery embolisation. The complications from both sites are outlined.

The paper entitled “*Radiofrequency ablation for treatment of symptomatic uterine fibroids*” is from the United States and the United Kingdom and reviews the use of radiofrequency ablation to treat fibroids. This is an excellent review detailing older now archaic methods and outlining the more modern safer techniques used in their setting.

Overall this journal is an excellent resource for physicians who wish to review uterine fibroids as well as all the methods available to treat them. The complications of fibroids are outlined in detail, and the different treatment modalities and possible complications are also outlined. This should become a standard compendium on fibroids for students and physicians for the benefit of increasing knowledge for examinations as well as the proper counseling and management of patients.

*Horace Fletcher* *Horace Fletcher* *Celia Burrell* *Celia Burrell* *Bharat Bassaw* *Bharat Bassaw* *Earlando Thomas* *Earlando Thomas*

